# Bidirectional variability in motor cortex excitability modulation following 1 mA transcranial direct current stimulation in healthy participants

**DOI:** 10.14814/phy2.12884

**Published:** 2016-08-05

**Authors:** Wolfgang Strube, Tilmann Bunse, Michael A. Nitsche, Alexandra Nikolaeva, Ulrich Palm, Frank Padberg, Peter Falkai, Alkomiet Hasan

**Affiliations:** ^1^ Department of Psychiatry and PsychotherapyLudwig Maximillian UniversityMunichGermany; ^2^ Department of NeurologyUniversity Medical Hospital BergmannsheilBochumGermany; ^3^ Leibniz Research Centre for Working Environment and Human Factors TU DortmundDortmundGermany

**Keywords:** Motor‐cortical plasticity, response variability, transcranial direct current stimulation

## Abstract

Due to the high interindividual response variability following transcranial direct current stimulation (tDCS), it is apparent that further research of the long‐lasting effects of the stimulation technique is required. We aimed to investigate interindividual variability following anodal tDCS and cathodal tDCS in a large‐scale prospective cross‐over study. Motor cortex physiology measurements were obtained using transcranial magnetic stimulation (TMS) in 59 healthy participants comparing motor‐evoked potential (MEP) magnitudes following two tDCS paradigms: 1 mA anodal tDCS for 13 min and 1 mA cathodal tDCS for 9 min. Analysis compared MEP changes over time for both polarities. Additionally, we applied hierarchical cluster analysis to assess the dynamics of poststimulation changes. Overall, anodal tDCS resulted in a significant increase in corticospinal excitability lasting for 40 min poststimulation, whereas cathodal tDCS did not alter corticospinal excitability. Cluster analysis revealed for cathodal tDCS both a cluster showing significant stable MEP reduction and a second cluster displaying MEP increase over time. Two diametrical clusters were also found for anodal tDCS. Regardless of polarity, individuals with MEP increase following stimulation showed steeper cortical recruitment curves compared to the clusters with decreased MEP magnitudes. The observed findings confirm a bidirectional modulation of corticospinal excitability following 1 mA tDCS in separate subgroups and the relationship to cortical recruitment.

## Introduction

Variability in long‐lasting motor‐cortex excitability changes induced by noninvasive brain stimulation (NIBS) techniques has been discussed for a long time, but recently gained more attention due to the first large‐scale publication on the efficacy and variability of theta‐burst stimulation provided by Hamada et al. (2013). Various determinants, including genetics, sex, age, anatomical features (cortical architecture and distance between stimulation electrode/coil and the brain), and physiological factors (individual recruitment of interneuron networks or prior history of synaptic activity), have been highlighted as potential aspects that may impact the efficacy of NIBS and the specific after‐effects observed (Ridding and Ziemann 2010; Hamada et al. 2013; Opitz et al. 2015; Hamada and Rothwell 2016).

To date many studies have reported significant intersubject response variability across literally all NIBS techniques (Muller‐Dahlhaus et al. 2008; Lopez‐Alonso et al. 2014; Wiethoff et al. 2014; Chew et al. 2015; Strube et al. 2015) and on average 30–50% of participants do not respond in the so‐called “expected direction”. For transcranial direct current stimulation (tDCS), the “expected direction” is defined as an increase in motor‐cortex excitability as indicated by increases in TMS‐elicited motor‐evoked potentials following anodal tDCS, whereas the opposite effect is expected following cathodal tDCS (Nitsche et al. 2008; Nitsche and Paulus 2011). These definitions of polarity‐dependent bidirectional changes have been derived from a multitude of experimental studies using tDCS with a current strength of 1 mA and a stimulation duration of 13 or 9 min for anodal and cathodal simulation respectively (Nitsche et al. 2008; Nitsche and Paulus 2011). However, many recent clinical and behavioral studies have applied higher current strengths (e.g. 2 mA) and longer stimulation durations (e.g. 20–30 min) assuming that these parameters might be more effective than the established parameters used in motor‐cortex physiology. One recently published crossover study following this line of reasoning applied 2 mA anodal or cathodal tDCS for 10 min to the left primary motor‐cortex of 53 healthy participants. Analyses revealed highly variable results and a subtle increase in MEPs following anodal tDCS but no decrease following cathodal tDCS (Wiethoff et al. 2014). Subsequent two‐step clustering analysis uncovered one cluster showing an increase in MEP amplitudes and one cluster for each stimulation polarity with no change in corticospinal excitability compared to baseline. Another study using stimulation parameters divergent from the standard showed that cathodal tDCS applied with 2 mA results in an increase in corticospinal excitability instead of the expected decrease (Batsikadze et al. 2013) offering one possible explanation for the aforementioned unexpected findings (Wiethoff et al. 2014). To further highlight the diversity of the after‐effects gained from NIBS techniques, research conducted by Lopez‐Alonso et al. compared 13 min of 1 mA anodal tDCS, PAS25 and intermittent theta‐burst stimulation in 56 healthy participants and could not establish an effect of any protocol on poststimulation corticospinal excitability compared to baseline (Lopez‐Alonso et al. 2014). Additional two‐step cluster analyses isolated one cluster showing increase and one cluster displaying decrease in corticospinal excitability for each of these excitability‐enhancing stimulation protocols (Lopez‐Alonso et al. 2014). In subsequent research the same group provided a longitudinal study conducted on 44 healthy participants revealing that anodal tDCS (13 min, 1 mA) increases corticospinal excitability for a period up to 30 min after stimulation, but not for a poststimulation observation period of 1 h (0–60 min) (Lopez‐Alonso et al. 2015). The intraindividual stability was relatively high with 56–78% percent of participants showing a congruent direction of excitability alteration following repeated intervention (Lopez‐Alonso et al. 2015). Finally, retrospective analyses of pooled data from several studies with small sample size, confirmed the bidirectional and polarity‐specific efficacy (~40% MEP increase after anodal and ~20% decrease following cathodal tDCS) of 1 mA tDCS in 85 healthy participants (Kuo et al. 2006). Supporting this finding a review of original data from three publications confirmed this polarity specific bidirectional manipulation of corticospinal excitability following 1 mA tDCS, but interestingly showed that the individual sensitivity to TMS, as measured by the intensity needed to elicited 1 mV MEPs, determines the amount of response to anodal tDCS (Labruna et al. 2016).

In the present study, we aimed to produce the first in depth investigation of interindividual variability of anodal and cathodal tDCS in a sample of 59 healthy participants using the standard parameters from motor cortex physiology studies: 1 mA anodal tDCS for 13 min and 1 mA cathodal tDCS for 9 min. These standard parameters have yielded stable and polarity‐specific after effects on corticospinal excitability (Nitsche and Paulus 2011), but have not been investigated regarding interindividual variability. Therefore, we applied those ‘classic’ tDCS protocols, which result in polarity‐dependent excitability alterations lasting for about 1 h. We chose these specific protocols because neuromodulatory tDCS effects show nonlinearity due to stimulation duration and intensity. In foregoing studies exploring variability of effects, protocols which applied different stimulation durations and intensities were used. This makes it difficult to decide if observed variability was caused by these specific protocol characteristics. We first hypothesized that the application of standard parameters (1 mA, 9–13 min) would result in more robust effects, as this standard configuration is discussed to be partially less sensitive to nonlinear intensity‐dependent effects (Monte‐Silva et al. 2010, 2013; Batsikadze et al. 2013). Second, based on the observations from other large‐scale studies, we further hypothesized that our results will be also subject to a significant intersubject variability. For the purpose of maintaining the comparability to previous publications (Hamada et al. 2013; Wiethoff et al. 2014), we used a two‐session cross‐over design with a related sample size and an after‐effect interval as well as a corresponding experimental setup.

## Methods

### Participants

After giving written informed consent, 59 healthy volunteers (mean age: 27.59 ± 7.72; 31 females; see Table 1), who were not experienced in the method, were consecutively recruited from the same geographical area. All participants underwent a standardized biographic interview and hand preference was assessed with the Edinburgh handedness inventory (Oldfield 1971). Participants with contraindication to TMS/tDCS or with a history of neurological or psychiatric illness were excluded. None of the participants had a history of alcohol/drug abuse and nobody was taking any neuroactive medication. Sociodemographic variables are presented in Table 1. The local medical ethics committee of the Ludwig‐Maximillians‐University of Munich approved the protocol and the study was conducted in accordance with the Declaration of Helsinki.

**Table 1:** Demographic variables of the sample

Variable	Frequency
Gender (female: male)	31: 28
Handedness (right: not right)	54: 5
Smoking (no: yes)	46: 13
	**Mean**
Age	27.59 ± 7.72
Education years	17.07 ± 2.96
Body weight (kg)	71.38 ± 17.68
Body height (cm)	174.20 ± 9.45
Body mass index (kg/m²)	23.36 ± 4.98

### TMS procedure and cortical excitability

During all experiments, participants were placed in a comfortable, half‐reclined sitting position with their head and arms at rest. We recorded electromyographic activity (EMG) via surface electrodes on the right first dorsal interosseus muscle (FDI). Raw signals were amplified and bandpass‐filtered (3 Hz–2 kHz range) using a Digitimer D‐360 amplifier setup (Digitimer Ltd, UK) and digitalized at 5 kHz using a 1401 data acquisition interface (Cambridge Electronic Design Ltd., Cambridge UK) controlled by Signal Software (Version 5, Cambridge Electronic design, Cambridge UK) (Hasan et al. 2012). At the end of the study, all data were analysed off‐line using the Signal Software. During the experiments, complete muscle relaxation was controlled by visual feedback of EMG activity.

As outlined elsewhere (Hasan et al. 2012), TMS was performed with a standard figure‐of‐eight coil (70 mm, The Magstim Company Ltd, UK) connected to a monophasic Magstim Bistim² stimulator (The Magstim Company Ltd, UK). In all experiments the coil was held tangentially to the skull above the left primary motor‐cortex (M1), with the handle pointing in a dorsolateral direction at a 45° angle from the midsagittal line leading to a posterior‐anterior directed current (Di Lazzaro et al. 1998). The stimulation site that produced the largest and stable motor evoked potential (MEP) at moderately suprathreshold stimulation intensities (“hot spot”) was marked with a skin marker for constant coil positioning.

### Transcranial direct current stimulation (tDCS)

We applied 1 mA tDCS through a battery‐driven constant current stimulator (NeuroConn GmbH, Ilmenau, Germany) and the current was applied through 7 × 5 cm saline‐soaked surface sponge electrodes. The target electrode was positioned over the representational field of the FDI as identified by TMS. The return electrode was contralaterally positioned above the right orbit. For anodal tDCS, we applied tDCS for 13 min and cathodal tDCS was applied for 9 min. We used a 15‐sec ramp‐up/down at the beginning and the end of the protocol to minimize any potential discomfort. This protocol has shown to be well suited for the induction of bidirectional cortical excitability changes (Nitsche and Paulus 2000, 2001; Nitsche et al. 2003b).

### Study design and cortical excitability measures

All participants underwent two experimental sessions (anodal vs. cathodal tDCS) on two different days in a randomized order. The interval between the first and second experiment was on average 7.42 (±2.33) days. Both sessions were conducted at the same time of day and the difference in the starting points of the two sessions was 0.97 h (±1.30). Four investigators performed the data collection and the complete two experimental sessions (anodal/cathodal) for each participant were always conducted by the same investigator. For all experiments, the same setup in the same laboratory was used. Sociodemographic data were recorded on the first study day and both experiments for a given participant were conducted by the same investigator. At baseline, resting motor thresholds (RMT) were determined according to standard publications (Rothwell 1997). The TMS intensity required to evoke MEPs of about 1 mV (S1 mV, peak to peak) was also recorded in the resting FDI muscle at baseline. Single pulse MEP measurements using the S1 mV intensity were conducted at baseline (40 stimuli) and after stimulation (timepoints 0, 5, 10, 20, 30 and 40 min; 20 stimuli at each timepoint) to monitor after‐effects following tDCS. Input–output curves (IO) were measured at baseline and 25 min after tDCS using increasing stimulus intensity order (90, 110 and 130% of RMT) with 7 stimuli for each intensity. In all experiments, TMS was applied at 0.2 Hz.

### Statistics

Statistical analyses were computed using SPSS 23 and level of significance was set at *α *= 0.05. To test for cortical excitability baseline differences between the two tDCS‐sessions of each participant, paired‐samples *t*‐tests were computed for all dependent variables (RMT, S1 mV, MEP amplitude). The timecourse of excitability changes (raw values of MEP amplitudes) before and after tDCS was explored with a repetitive‐measures analysis of variance (RM‐ANOVA) (7 × 2) with TIME (baseline, 0, 5, 10, 20, 30, and 40 min) and CONDITION (anodal, cathodal) as within‐subject factors. Input–output curves were compared before and after tDCS with a RM‐ANOVA including the within‐subject factors TIME (before and after tDCS) and INTENSITY (RECR90%, RECR110% and RECR130% RMT) separately for the anodal and cathodal tDCS experiment. We then performed agglomerative hierarchical cluster analysis (HCA) on the individual MEP courses (variables: array of raw MEP values for all seven data measurement timepoints: baseline and post 0, 5, 10, 20, 30, and 40 min) in order to identify subgroups with consistently dissimilar MEP courses. For HCA, we used the algorithms embedded in SPSS (see below). We chose the agglomerative approach of clustering as this algorithm organizes participants’ MEP courses according to their similarity, resulting in separate hierarchically structured clusters (=subgroups). Squared Euclidean distance was used as interval measure and Ward's method was set as the criterion for choosing which MEP courses to merge in each cluster at each step as it ensures that participants with highly similar MEP courses are assigned to the same clusters (for an overview please see, Murtagh and Legendre 2014; Yim and Ramdeen 2015). The range of solutions (i.e. the maximal numbers of clusters identified by the algorithm) was defined between 2 and 4 to enable the algorithm to cluster more than two subgroups if no clear separation between two distinct subgroups could be identified in the sample. We decided to accept the 2‐solution decision as only this decision resulted in clusters with comparable sample‐sizes and used the SPSS function for cluster‐coding. This HCA analysis was performed separately for both the anodal and cathodal tDCS experiments and obtained two unique clusters for both stimulation conditions. These clusters were then post hoc included as fixed factors (called CLUSTER) in subsequent analyses: First, baseline excitability and demographic data were compared between the two given clusters of each stimulation session with two‐tailed independent t‐tests or χ^2^ tests. Second, mixed‐factorial RM‐ANOVAs with the within‐subject factor TIME and the between‐subject factor CLUSTER were computed for anodal and cathodal tDCS separately. Finally, a mixed‐factorial RM‐ANOVA for both tDCS sessions with the within‐subject factors TIME and INTENSITY and the between‐subject factor CLUSTER was calculated to compare differences of recruitment curves between the clusters before and after tDCS. Based on previous publications (Hamada et al. 2013; Wiethoff et al. 2014), response to tDCS was assessed by the grand average (GA) of normalized MEPs (0 to 40 min). Distribution of responder and cluster membership were compared with descriptive statistics. For RM‐ANOVAs, sphericity was tested with the Mauchly's test and, if necessary (Mauchly's test < 0.05), Greenhouse–Geisser correction was applied. In the case of significant interactions in the RM‐ANOVAs, Least Significant Difference (LSD) tests were conducted for within‐group comparisons and independent‐samples *t*‐tests for between‐group comparisons over time (all two‐tailed, *P* < 0.05). Pearson correlations (two‐tailed) were performed between physiological baseline values (RMT, S1 mV, MEP, RECR90%, RECR110%, RECR130%) with the averaged MEP values after anodal and cathodal tDCS (mean 0–40 min). Post hoc t‐tests were not corrected for multiple comparisons. Data are presented as mean ± standard deviation unless otherwise indicated. All figures, apart from the response distribution, represent raw data.

## Results

Demographic variables are shown in Table 1. No baseline differences in excitability were observed between both conditions (see Table 2). To test whether participants had a shift of baseline excitability, the first 20 MEPs were compared to the last 20 MEPs of the baseline measure. This analysis did not reveal significant differences for the anodal (*P* = 0.618) and cathodal (*P* = 0.143) experiment.

**Table 2:** Baseline physiological measures

	Anodal	Cathodal	*P* values
RMT [%]	34.98 ± 7.00	34.27 ± 7.31	0.158
S1 mV [%]	42.20 ± 9.14	41.83 ± 9.90	0.543
Baseline MEP size [mV]	1.17 ± 0.34	1.16 ± 0.30	0.910

RMT, resting motor threshold; S1 mV, stimulus intensity to elicit 1 mV MEP; MEP, motor‐evoked potential.

All data are presented as mean ± standard deviation.

### Overall effects of tDCS

**Figure 1:**
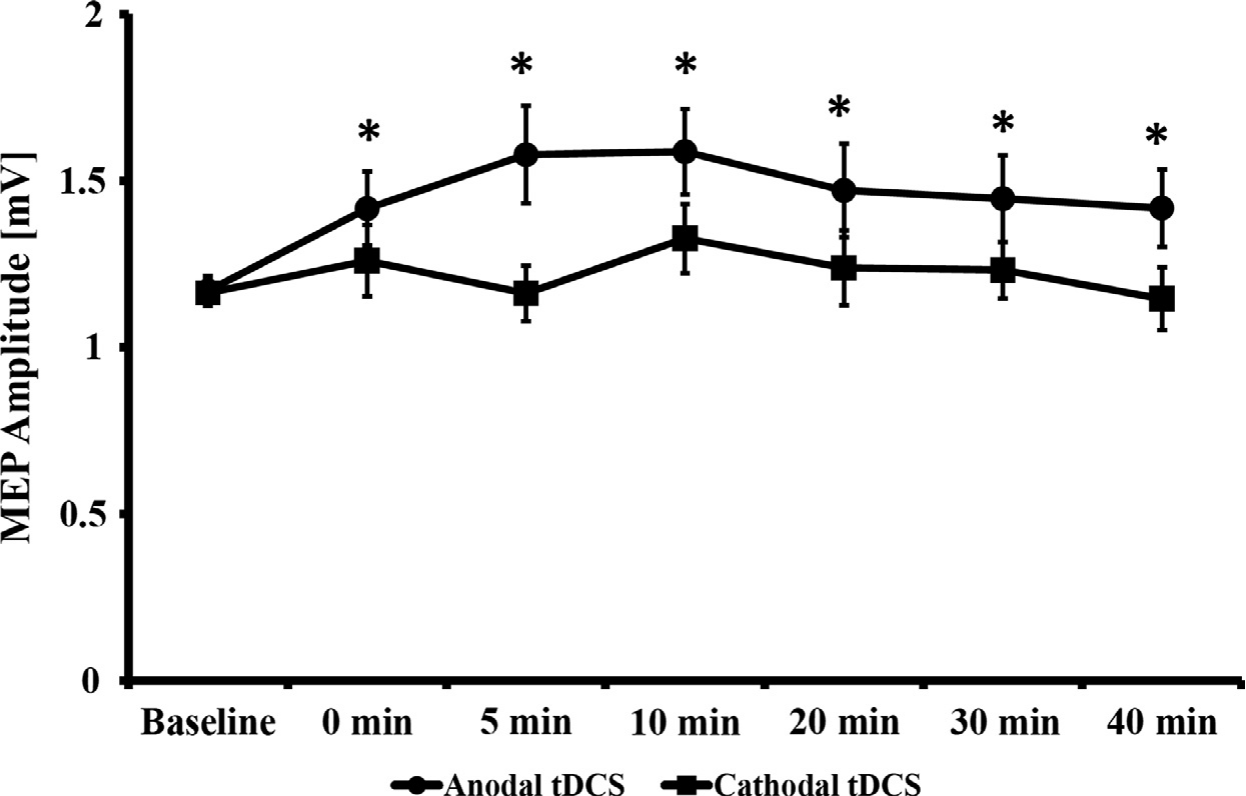
MEP changes over time. Asterisks indicate significant differences (Least significant difference) between baseline and the respective time point. All data are presented as mean ± standard error of the mean.

Repetitive‐measures analysis of variance revealed a significant effect of CONDITION (*F*_(1, 58)_ = 5.004; *P* = 0.029) and a significant effect of TIME (*F*_(4.4, 255.2)_ = 2.720; *η*² = 0.045; *P* = 0.026; observed post hoc power = 0778), but no CONDITION × TIME interaction (*F*_(4.7, 272.9)_ =1.432; *P* = 0.216). For anodal tDCS, ANOVA showed a significant effect of TIME (*F*_(4.4, 253.1)_ = 2.607; *P* = 0.032), whereas this analysis showed no significance for cathodal tDCS (*F*_(4.3, 251.9)_ = 1.114; *P* = 0.352). These results indicate an overall increasing effect of anodal tDCS on MEP magnitudes, but no significant excitability shifts following cathodal tDCS (see Fig. 1). RM‐ANOVA results survived the addition of the factor ‘investigator’ (*F*_(3, 55)_ = 0.599, *P* = 0.618) with the main effect CONDITION (*F*_(1, 55)_ =4.612; *P* = 0.036) and TIME (*F*_(4.3, 238.7)_ = 2.462, *P* = 0.041) still being significant and not showing a significant CONDITION × TIME × INVESTIGATOR interaction (*F*_(13.8, 252.2)_ = 0.869, *P* = 0.592). Post hoc LSD tests showed a significant MEP increase after anodal tDCS compared to baseline at all post‐tDCS time points (all‐*P* between 0.01 and 0.049). For cathodal tDCS, no post hoc tests were performed in the ANOVA. For anodal/cathodal tDCS, RM‐ANOVA of input–out curves showed a significant effect of INTENSITIY (*F*_(1.35, 71.46)_ = 96.848; *P* < 0.001/*F*_(1.19, 67.62)_ = 120.228; *P* < 0.001), but no effect of TIME (*F*_(1, 53)_ = 0.117; *P* = 0.733/*F*_(1, 57)_ = 0.702; *P* =0.405) and no TIME x INTENSITY interaction (*F*_(1.71, 90.57)_ =2.139; *P* = 0.131)/(*F*_(1.31, 74.74)_ = 0.702; *P* = 0.442).

### Clustering analysis

**Figure 2:**
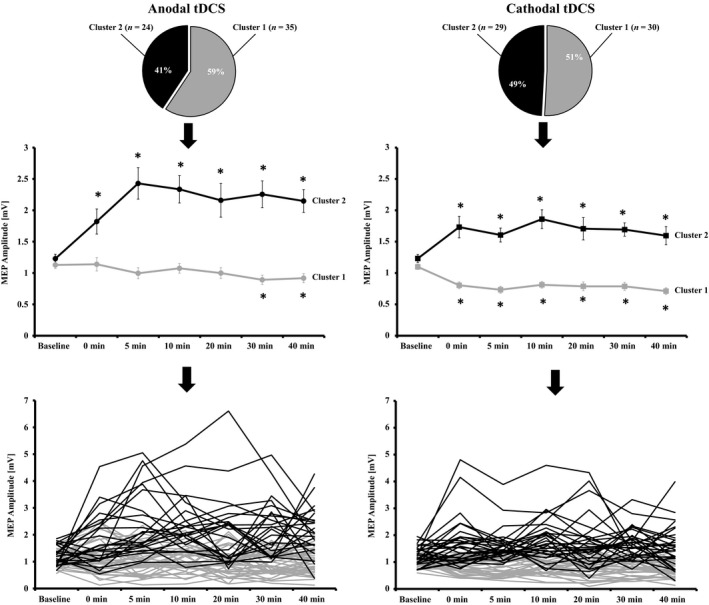
Cluster distribution and MEP changes over time. For anodal tDCS, one cluster with an increase in corticospinal excitability following stimulation (cluster 2) and one cluster (cluster 1) with no excitability change/slight decreases could be detected. For cathodal tDCS, one cluster (cluster 2) with increases following intervention and a cluster (cluster 1) with a decrease of corticospinal excitability could be detected. Asterisks indicate significant differences (Least Significant Difference) between baseline and the respective time point. Individual data presentation indicates for anodal and cathodal tDCS large intersubject variability, but also shows the grouping of individual subjects to the respective clusters 1 or 2. All data are presented as mean ± standard error of the mean.

We then used agglomerative hierarchical clustering to detect subgroups within the aforementioned sample. We were able to detect two clusters for anodal tDCS (cluster 1: *n* = 35; cluster 2: *n* = 24) and two clusters for cathodal tDCS (cluster 1: *n* = 30; cluster 2: *n* = 29). Apart from a subtle difference in education years between clusters for cathodal tDCS, no significant sociodemograpic or baseline excitability differences were detected between both clusters of anodal or cathodal tDCS, respectively (see Table 3). For anodal tDCS, a mixed‐factorial RM‐ANOVA showed a significant effect of TIME (*F*_(4.18, 238.11)_ = 4.724; *P* = 0.001), a significant effect of CLUSTER (*F*_(1, 57)_ = 66.226; *P* < 0.0001) and a significant TIME × CLUSTER interaction (*F*_(4.18, 238.11)_ = 8.356; *P* < 0.0001). For cathodal tDCS, no significant effect of TIME (*F*_(4.20, 239.49)_ = 1.275; *P* = 0.279), but a significant effect of CLUSTER (*F*_(1, 57)_ =68.897; *P* < 0.0001) and a significant TIME × CLUSTER interaction (*F*_(4.20, 239.49)_ = 6.699; *P* < 0.0001) was observed. ANOVA for anodal‐cluster 1 showed a significant effect of TIME (*F*_(6, 204)_ = 2.263; *P* = 0.039) and post hoc LSD tests showed a significant MEP decrease after 30 and 40 min (*P* = 0.011; *P* = 0.024), but no effects at the other poststimulation time points (all *P* ≥ 0.185). For anodal‐cluster 2, a significant effect of TIME (*F*_(3.53, 81.16)_ =5.439; *P* = 0.001) was revealed and post hoc LSD tests showed a significant MEP increase for all poststimulation time bins (all *P* = 0.013 to <0.001). ANOVA for cathodal‐cluster 1 revealed a significant effect for TIME (*F*_(6, 174)_ = 8.306; *P* < 0.0001) and posthoc LSD test showed a significant MEP decrease for all poststimulation time bins (all *P* < 0.001). Finally, analyses for cathodal‐cluster 2 revealed a significant effect for TIME (*F*_(3.86, 108.11)_ = 3.172; *P* = 0.018) and post hoc LSD test showed a significant MEP increase in all poststimulation time bins (all *P* = 0.018 to <0.001) (see Fig. 2).

**Table 3:** Comparison of demographic variables and physiological baseline measures between clusters

	Cluster 1	Cluster 2	*P* values
Anodal tDCS			
Demography			
Gender (female: male)	16: 19	15: 9	0.205
Handedness (right: not right)	31: 4	23: 1	0.325
Smoking (no: yes)	28: 7	18: 6	0.649
Age	27.83 ± 8.42	27.25 ± 6.73	0.780
Education years	17.29 ± 3.13	16.75 ± 2.73	0.499
Body weight (kg)	70.01 ± 16.17	73.38 ± 19.86	0.477
Body high (cm)	172.97 ± 8.71	176.00 ± 10.36	0.230
Body mass index (kg/m²)	23.29 ± 4.59	23.48 ± 5.60	0.885
Physiology			
RMT [%]	34.80 ± 7.49	35.25 ± 6.38	0.811
S1 mV [%]	41.89 ± 9.82	42.67 ± 8.22	0.750
Baseline MEP size [mV]	1.13 ± 0.34	1.23 ± 0.35	0.271
Cathodal tDCS			
Gender (female: male)	15: 15	16:13	0.691
Handedness (right: not right)	26: 4	28: 1	0.173
Smoking (no: yes)	23: 7	23: 6	0.807
Age	26.40 ± 7.16	28.83 ± 8.21	0.231
Education years	16.32 ± 2.23	17.84 ± 3.43	0.046*
Body weight (kg)	72.47 ± 18.21	70.24 ± 17.37	0.632
Body high (cm)	174.20 ± 9.03	174.21 ± 10.02	0.998
Body mass index (kg/m²)	23.80 ± 5.36	22.91 ± 4.60	0.499
Physiology			
RMT [%]	35.20 ± 8.40	33.31 ± 5.98	0.323
S1 mV [%]	42.80 ± 11.49	40.83 ± 8.02	0.449
Baseline MEP size [mV]	1.10 ± 0.24	1.23 ± 0.34	0.090

RMT, resting motor threshold; S1 mV, stimulus intensity to elicit 1 mV MEP; MEP, motor‐evoked potential. *P < 0.05.

All data are presented as mean ± standard deviation.

### Input–output curves after clustering

**Figure 3:**
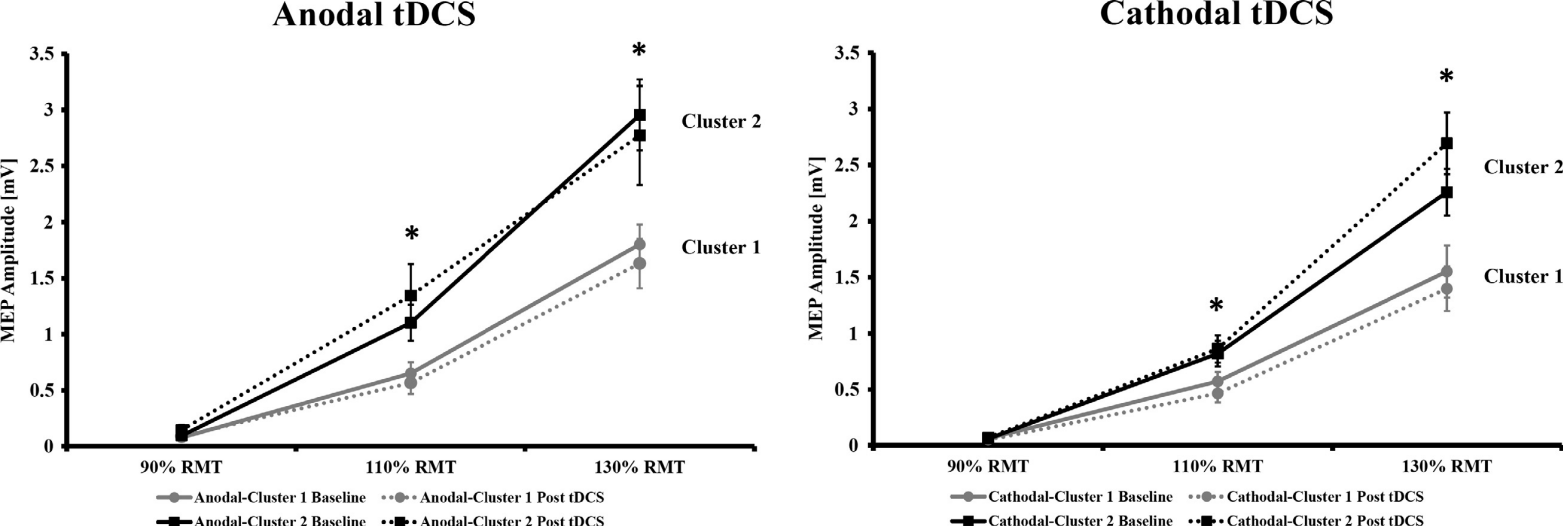
Input–output curves (cortical recruitment) before and after tDCS separated for polarity and cluster membership. Asterisks indicate significant differences (independent t‐test) between cluster 1 and 2 for a given intensity. All data are presented as mean ± standard error of the mean.

For anodal tDCS, mixed‐factorial RM‐ ANOVA for I/O curves showed a significant effect for INTENSITY (*F*_(1.38, 71.84)_ = 118.585; *P* < 0.0001), for CLUSTER (*F*_(1, 52)_ = 13.073; *P* = 0.001) and for the INTENSITY × CLUSTER interaction (*F*_(1.38, 71.84)_ = 8.406; *P* = 0.002), but no further main effects or interactions (all *P* ≥ 0.101). At baseline and after anodal tDCS, higher MEP values for 110% (df =37.99, *P* = 0.010; df = 28.06, *P* = 0.006) and 130% RMT (df = 53, *P* = 0.001; df = 28.67, *P* = 0.015), but not for 90% RMT (df = 57, *P* = 0.743; df = 56, *P* = 0.232) were observed for anodal‐cluster 2 compared to anodal‐cluster 1. Within each cluster, no differences between baseline and post‐tDCS I/O curves at all intensities were observed (all *P* ≥ 0.109). For cathodal tDCS mixed‐factorial RM‐ANOVA for I/O curves showed a significant effect for INTENSITY (*F*_(1.21, 67.95)_ = 140.021; *P* < 0.0001), for CLUSTER (*F*_(1, 56)_ = 12.265; *P* = 0.001), for the INTENSITY × CLUSTER (*F*_(1.21, 67.95)_ = 9.228; *P* = 0.002), for TIME × CLUSTER (*F*_(1, 56)_ = 5.542; *P* = 0.022), a trend for the INTENSITY × TIME × CLUSTER interaction (*F*_(1.33, 74.50)_ =3.496; *P* = 0.053), but no further main effects or interaction (all *P* ≥ 0.345). At baseline and after cathodal tDCS, higher MEP values for 110% (df = 57, *P* = 0.023; df = 48.53, *P* = 0.009) and 130% RMT (df = 56, *P* = 0.031; df = 56, *P* < 0.001), but not for 90% RMT (df = 57, *P* = 0.858; df = 57, *P* = 0.143) were observed for cathodal‐cluster 2 compared to cathodal‐cluster 1. Within each cluster, apart from a trend‐level difference for cathodal‐cluster 2 at 130% RMT (*P* = 0.056), no differences between baseline and post‐tDCS I/O curves at all intensities were observed (all other *P* ≥ 0.134) (see Fig. 3).

### Responder analyses

**Figure 4:**
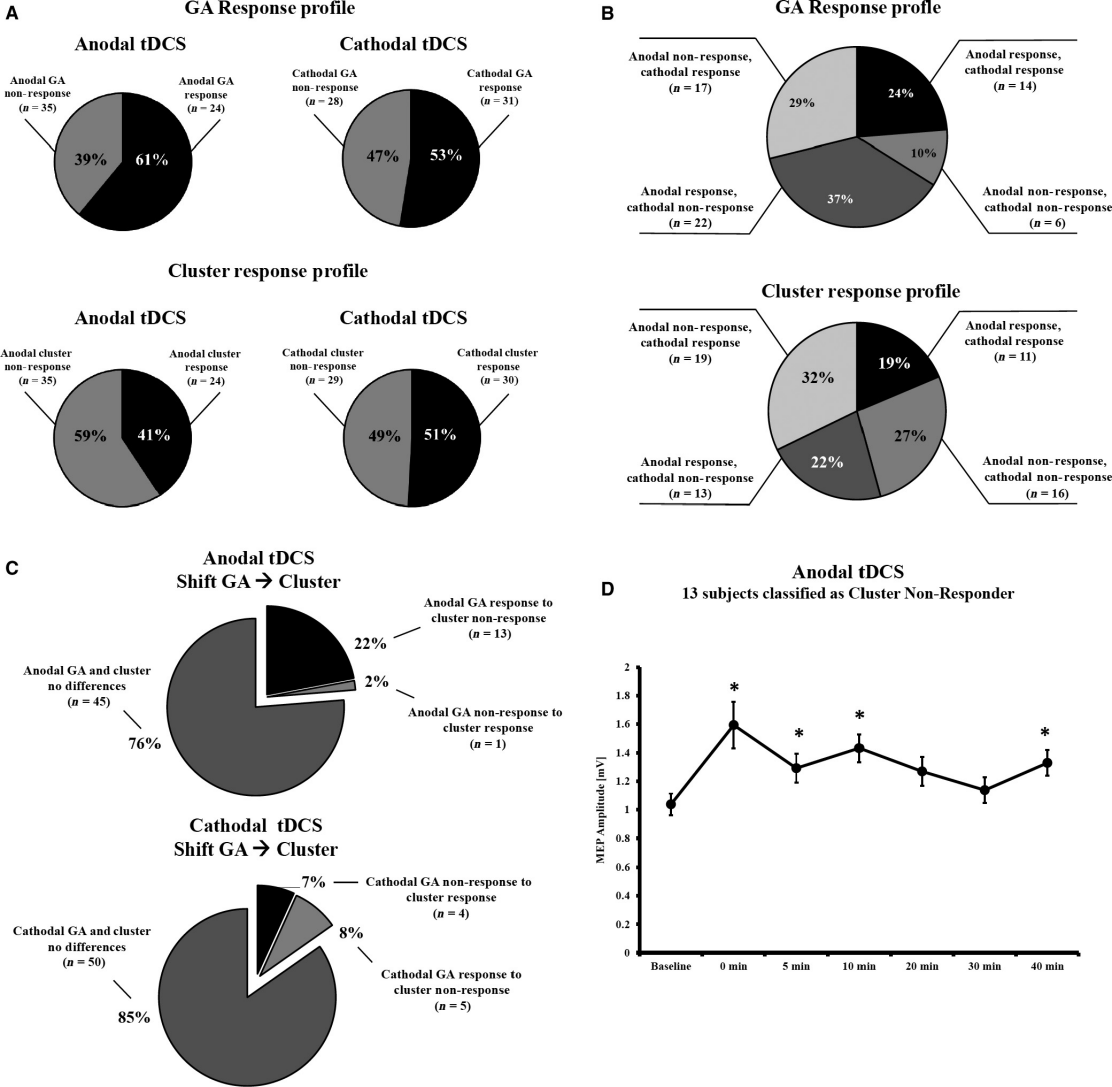
Distribution of responders and nonresponders. (A) Frequency distribution of GA and clustering responders and nonresponders for both stimulation polarities; (B) Response profile of tDCS taking into account the response pattern for both stimulation polarities; (C) Distribution of participants who differed in the response profile comparing the GA method with the clustering method; (D) MEP changes over time for those 13 participants who were GA responders following anodal tDCS, but who were clustered into the cluster 1. Asterisks indicate significant differences (Least Significant Difference) between baseline and the respective time point. All data are presented as mean ± standard error of the mean.

Using the response criteria from previous papers based on the grand average (GA) normalized to the baseline (Hamada et al. 2013; Wiethoff et al. 2014), we had 61% responders and 39% nonresponders in the anodal experiment, whereas the frequencies were 53% responders and 47% non‐responders in the cathodal group. In the anodal group, 23 of 24 cluster 2 members were also GA responders, whereas 1 cluster 2 member was GA nonresponder. Here 13 of 35 cluster 1 members were GA responders and the remaining 22 cluster 1 members were GA nonresponders. In the cathodal group, 26 of 30 cluster 1 members were GA responders, whereas the remaining 4 were GA nonresponders. 24 of cluster 2 members were GA nonresponders and the remaining 5 cluster 2 members were GA responders. We then compared the response profiles using the GA and clustering method (see Fig. 4A–C) confirming a higher overlap between both methods in the cathodal compared to the anodal group. As a next step, we analyzed the overlap between both classification methods to identify those participants who were classified to different response profiles comparing the shift from GA to Clustering classification. For cathodal tDCS, 85% were classified with both methods in the same manner, whereas this value was 76% in the anodal group. A group of 13 participants (22%) were classified as anodal GA‐responders, but Clustering‐nonresponders, we analyzed the MEP course of these participants. ANOVA showed a significant effect of TIME (*F*_(6, 72)_ = 3.244; *P* = 0.007) and post hoc LSD tests showed an MEP increase after 0, 5, 10, and 40 min (*P* = 0.002 to 0.023, all other *P* ≥ 0.158) (see Fig. 4D).

### Correlations analyses

Finally, we analyzed the correlation between baseline variables (RMT, S1 mV, MEP, RECR90%, RECR110%, RECR 130%) with the averaged MEP values after anodal and cathodal tDCS. Using a significance threshold of 0.0083 (for six comparisons), only baseline RECR110% (*P* < 0.001, *r* = 0.498) baseline RECR130% (*r* < 0.001, *r* = 0.539) correlated significantly with the average MEP values following anodal tDCS (all other *P* ≥ 0.076). For cathodal tDCS, baseline RECR110% (*P* < 0.001, *r* = 0.476) and baseline RECR130% (*P* = 0.004, *r* = 0.370) correlated significantly with the average MEP values following cathodal tDCS. Baseline MEPs (*P* = 0.0085) showed significant correlations that would not survive adjustment for 6‐factor comparisons and all other correlations were not significant (all *P* ≥ 0.060).

## Discussion

The results of this tDCS study provide further evidence for a high intersubject variability of NIBS applied to the human motor cortex. To the best of our knowledge, this is the first large‐scale prospective cross‐over study investigating the after‐effects of 13 min anodal and 9 min cathodal tDCS administered at an intensity of 1 mA. Anodal tDCS resulted in a significant increase in corticospinal excitability that lasted for the whole poststimulation period of 40 min, as expected from the results of early motor‐cortex tDCS studies (Nitsche and Paulus 2001; Liebetanz et al. 2002; Nitsche et al. 2003a) that have been confirmed and replicated over the years (for review see (Nitsche and Paulus 2011). Contrary to our expectations, supported by the aforementioned studies, cathodal tDCS did not alter corticospinal excitability (whole‐group analysis).

For anodal tDCS, other large‐scale studies have that shown variable results. Lopez‐Alonso et al. did not observe an overall effect of anodal tDCS (13 min, 1 mA) in a sample of 56 healthy participants (Lopez‐Alonso et al. 2014), whereas at a sample‐size of 45 participants, a significant increase in corticospinal excitability was observed for the first 30 min poststimulation (Lopez‐Alonso et al. 2015). Wiethoff et al. used different stimulation parameters for anodal tDCS and showed again a significant increase in MEP amplitudes following anodal tDCS (Wiethoff et al. 2014). There is no large‐scale study for cathodal tDCS available, that used the here applied standard parameters (9 min, 1 mA), but our results showing no change in corticospinal excitability after cathodal tDCS (whole group analysis) overlap with the findings from previous studies that have utilized 10 min of tDCS with 2 mA (Wiethoff et al. 2014).

The percentages of responders vs. nonresponders in our study according to the GA definition were 61%/39% for anodal and 53%/47% for cathodal tDCS, whereas Wiethoff et al. (2014) reported 75%/25% after anodal and 60%/40% after cathodal tDCS and Lopez‐Alonso et al. reported a GA response of 50% after anodal tDCS (Lopez‐Alonso et al. 2014). This distribution is not in line with previous tDCS studies using our stimulation parameters, but confirms the observation of a high intersubject variability following NIBS techniques (Muller‐Dahlhaus et al. 2008; Hamada et al. 2013; Lopez‐Alonso et al. 2014). The application of the GA method to categorize the continuous poststimulation data into the categorical variables has several practical advantages, but ignores the dynamics of poststimulation changes in corticospinal excitability. Therefore, we decided to perform an agglomerative hierarchical cluster analysis on the raw MEP data before and after stimulation, as this clustering method does not need a priori information about the required cluster. Additionally, we used the raw data rather than normalized data to account for the variability and impact of the MEP baseline in the model. This analysis revealed two clusters for anodal tDCS. One cluster was characterized by constant corticospinal excitability that turned to a subtle decrease after 30 and 40 min, whereas the second cluster showed a steep increase in corticospinal excitability within the first 10 minutes post‐tDCS that remained stable. For cathodal tDCS two clusters were also identified; the first cluster showed a significant MEP size reduction that remained stable over the whole poststimulation period, whereas the second cluster showed a significant MEP increase over time. Comparison of our anodal clusters with clusters derived from two‐step cluster analyses by other groups that used the same stimulation parameters (Lopez‐Alonso et al. 2014) displays a related pattern, but only an overlap for the cluster showing increases in corticospinal excitability in the 2 mA study (Wiethoff et al. 2014). The latter study showed for 2 mA cathodal tDCS, one cluster with increased MEP magnitudes and one neutral cluster with no change in corticospinal excitability, which contrasts our finding of a robust cluster displaying decreased MEP magnitudes following 1 mA cathodal tDCS. Therefore, our study confirms for the first time robust subgroups that show a bidirectional modulation of corticospinal excitability following 1 mA tDCS based on a clustering method without a priori definition of outcome. The overlap of cluster membership and GA response group was relatively stable for cathodal tDCS. However, for anodal tDCS this was only the case for the subgroup showing an increase in corticospinal excitability following stimulation. Therefore, it may be speculated whether the anodal nonresponder group falls into two subgroups, a neutral one and one showing excitability decreases. Further analyses indicate that for cathodal tDCS 85% of all participants were classified using the GA‐method or an independent hypothesis‐free clustering method in the same manner, whereas this is only true for 76% of all anodal datasets. For the 13 participants who were nonresponders according to the clustering method, we here observed that they are identified as anodal tDCS responders by the GA method. Subsequent post hoc analyses then showed that this subgroup also showed an increase in corticospinal excitability following stimulation. Hence, one could speculate that for anodal tDCS larger sample sizes might have revealed a third cluster in between the obtained clusters with high excitability increases and neutral response or excitability decreases following stimulation. Importantly, both clusters of each polarity did not differ regarding baseline characteristics of excitability, excluding that these factors determine differences in excitability modulation between groups.

Regardless of anodal or cathodal stimulation, the individuals who fell into the cluster for an increase in excitability showed steeper input–output curves before and after stimulation compared to the clusters displaying a decrease of corticospinal excitability. Input–output curves can be considered as index of global cortico‐spinal excitability reflecting the strength of corticospinal projections (Devanne et al. 1997; Abbruzzese and Trompetto 2002). The slope of the input–output curve has been linked to the recruitment of larger neuronal populations (Chen 2000; Abbruzzese and Trompetto 2002; Nitsche et al. 2005). The corresponding increase in the input–output slope observed in the clusters with an increase in excitability might therefore indicate that this group has a higher probability of motor neuron firing or that the TMS pulse recruits more motor neurons (Devanne et al. 1997; Moller et al. 2009). This idea is also supported by the finding that participants with larger MEPs in the recruitment curves (110%, 130% RMT) have higher mean MEP values after tDCS. However, we measured the input‐output curve only with increasing, but not with decreasing sequence, we did not use high stimulation‐intensities required to reach a stable plateau (Moller et al. 2009) and we applied only a limited number of trials per intensity. Therefore, the link between steepness of input‐output curve and the likelihood to develop increases in corticospinal excitability following tDCS should be interpreted prudently and more research is needed to confirm this relationship. Moreover, we were not able to detect differences in slopes of the input–output curves before and after tDCS (Nitsche et al. 2005), which needs to be considered as further limitation of these measures. One possible reason for this finding could be the relatively limited number of trials per intensity.

Wiethoff et al. showed a correlation between small baseline MEPs and the likelihood to develop an increase in corticospinal excitability. In our sample, we observed the opposite effect. The correlations between baseline MEPs and mean post‐MEPs were *r*² = 0.054 (*P* = 0.076) for anodal and *r*²=0.115 (*P* = 0.008) for cathodal tDCS, indicating that those participant with higher baseline have an increased likelihood for increases of excitability. However, baseline MEPs did not differ between clusters showing an increase or decrease in corticospinal excitability and the correlation was uncorrected for multiple comparisons. Despite these limitations, this finding has been corroborated by a recently published paper indicating that participants who are more sensitive to TMS (indicated by low S1 mV) have an increased likelihood to show an increase in excitability, whereas such a relationship could not be established for cathodal tDCS (Labruna et al. 2016). More research is needed to disentangle the relationship between baseline MEP amplitudes and the likelihood to develop a plasticity response in a certain direction.

Current tDCS protocols are applied for all participants of a study with the same configuration regarding stimulation intensity and duration, whereas TMS‐based plasticity protocols adapt the intensity to the individual resting motor threshold, or other predefined and individualized intensities. We know that the individual TMS motor threshold is related to the coil‐cortex distance (McConnell et al. 2001; Herbsman et al. 2009) and for tDCS, modeling studies indicate that cortical electric field density is a function of the applied current intensity that is at least in part related to the anatomical condition (Datta et al. 2012; Opitz et al. 2015). Therefore, two further hypothetical explanations for the reported variability and clusters following tDCS appear to be possible. First, it may be speculated that different physiological and anatomical properties of the participating participants resulted in an *ineffective stimulation*. However, as we observed clusters for each condition with a significant change in cortico‐spinal excitability, and as studies using low range intensities of <1 mA can also produce excitability changes (Nitsche and Paulus 2000; Bastani and Jaberzadeh 2013; Kidgell et al. 2013; Vaseghi et al. 2015), this possibility seems to be unlikely. Secondly, one could speculate whether individual anatomical or physiological differences across participants results in *nonlinear stimulation effects*. For example, the application of cathodal tDCS with 2 mA stimulation has resulted in an increase rather than decrease in corticospinal excitability (Batsikadze et al. 2013), and the use of 1 mA cathodal tDCS in children (age <14 years), who have a different cortical anatomy than adults, also exhibited enhanced excitability (Moliadze et al. 2015). Thus, one could speculate whether current flow results in higher cortical current in the cathodal cluster 2 members with the result of reversed after‐effects. For anodal tDCS, we were able to find an overall increase in MEP amplitudes indicating that the aforementioned effect might be less pronounced. This is supported by the observation that also higher and lower current intensities result in a robust excitability enhancement following anodal tDCS (Bastani and Jaberzadeh 2013; Batsikadze et al. 2013; Wiethoff et al. 2014). However, also for anodal tDCS, we were able to detect a cluster with a slight decrease of corticospinal excitability over time. In principal accordance, nonlinear excitability‐reducing effects have been induced by prolonged anodal current flow [26 min (Monte‐Silva et al. 2013)], and under medication, which enhances intracellular calcium concentration or NMDA receptor activation (Thirugnanasambandam et al. 2011; Lugon et al. 2015). In these conditions, reduction of calcium influx abolished the respective excitability diminution, or resulted in recovery of excitability enhancement (Monte‐Silva et al. 2013; Lugon et al. 2015). Thus, one might speculate that similar to the situation in cathodal tDCS, the anodal tDCS nonresponder group also exhibited different effects compared to the responders due to calcium‐dependent mechanisms; however, this hypothesis awaits future empirical testing.

Most probably, a multitude of determinants underlie the observed variability (Ridding and Ziemann 2010) and there is a need to disentangle the interplay between the underlying various intrinsic and extrinsic factors (Ridding and Ziemann 2010; Hordacre et al. 2015; Hamada and Rothwell 2016). In this context, one should take into account interindividual variability and also the heterogeneity of plasticity responses, which is observed even when many intrinsic and extrinsic factors are strictly controlled [e.g., rat hippocampal slice cultures (Debanne et al. 1999)]. NIBS have to be considered as neuromodulatory interventions, which critical depend on brain state and trait characteristics in a nonlinear fashion. Therefore, interindividual variability is not surprising and the fact that these effects have a relatively high intraindividual stability (Lopez‐Alonso et al. 2015) supports the evidence that the after effects are real physiological effects.

### Limitations

We chose the relatively larger electrode sizes of 7 × 5 cm as they are the most commonly used ones in the majority of tDCS studies reporting polarity‐dependent MEP alterations and therefore establish adequate comparability between these studies and our results. Additionally, equal results have been obtained by studies investigating tDCS after‐effects over M1, using smaller electrodes [e.g. (Nitsche et al. 2007)]. It has to be taken into account however, that with increasing size of the used tDCS electrodes neighbouring regions such as the premotor cortex might be affected by stimulation. And although premotor tDCS does not result in direct MEP magnitude changes elicited over the primary motor cortex (Boros et al. 2008) potential effects of tDCS on motor cortex afferents need to be considered. One further methodological limitation arises from the fact that we assessed cortical activity changes solely by measuring motor cortex excitability and that we did not include behavioral measures like motor learning. At the same time, the activity modulations induced by tDCS affect most likely various additional regions, such as the supplementary motor area and the prefrontal cortex. Hence, the potential extend by which other adjacent brain regions might contribute to the observed MEP magnitude changes and variability following both anodal and cathodal tDCS is yet unknown. However, the M1 region is regarded as a suitable model system for cortical plasticity studies (Nitsche and Paulus 2011). Further, the observed variability following tDCS might have been enhanced in the case of inexperienced participants, who might have been more anxious and may have had difficulties to relax completely and to stay in a constant state of relaxed alertness as experienced participants would do (Woods et al. 2016).

## Conclusions

Using standard physiological configuration and stimulation parameters (1 mA, 13 min/9 min), we showed for the first time that motor‐cortex excitability modulation following anodal and cathodal tDCS is also subject to interindividual variability. The findings of the current study are strengthened by the use of a sufficient sample‐size, a naturalistic design and inclusion of participants unexperienced with the method, to reduce the likelihood of an enrichment of responders. Future studies will have to further identify relevant factors underlying the interindividual variability and will have to focus on the prediction of responders and nonresponders.

## Conflicts of interest

The authors declare that except income received from primary employers, no financial support or compensation has been received. However, other conflicts of interests not related to this publication are as follows: W. Strube has received paid speakership by Mag and More; T. Bunse reports no conflicts of interest; M.A. Nitsche is a member of the advisory boards of Neuroelectronics, UCB, and Eisai, and was honorary speaker for UCB, Eisai, and Glaxo‐SmithKline; A. Nikolaeva reports no conflicts of interest; U. Palm reports no conflicts of interest; F. Padberg has received speakers honorarium from Mag&More GmbH, and material support from neuroConn GmbH, Ilmenau, Germany, and Brainsway Inc., Jerusalem, Israel; P. Falkai was a member of the advisory boards of Janssen‐Cilag, AstraZeneca, Eli Lilly, and Lundbeck. A. Hasan has been invited to scientific meetings by Lundbeck, Janssen‐Cilag, and Pfizer, and he received paid speakership by Desitin, Otsuka and BAK. He is a member of the Roche Advisory Board.
